# Potential Active Compounds of *Ganoderma lucidum* and Their Anticancer Effects: A Comprehensive Review

**DOI:** 10.1002/fsn3.70741

**Published:** 2025-08-01

**Authors:** Yuan Liu, Sizhu Ren, Qing Sang, Xi Cheng, Yanmeng Bi

**Affiliations:** ^1^ College of Integrated Traditional Chinese and Western Medicine Jining Medical University Jining China; ^2^ College of Life Sciences Langfang Normal University Langfang China

**Keywords:** cancer, functional ingredients, *Ganoderma lucidum*, polysaccharides, triterpenoids

## Abstract

The incidence and mortality rates of cancer patients continue to rise. Although many physical and chemical therapies, such as targeted tumor treatments, are effective against cancer, they are often associated with varying degrees of side effects. Chinese herbal medicine, as an alternative therapy, can effectively alleviate clinical symptoms of cancer patients by preventing or stabilizing tumor growth. *Ganoderma lucidum* is a medicinal fungus that contains a variety of bioactive compounds in its fruiting body and mycelia. The importance of *G. lucidum* as a source of functional ingredients has been well recognized. As a result, the isolation and investigation of novel bioactive ingredients from *G. lucidum* have attracted considerable attention. Functional ingredients identified from this fungus, such as natural polysaccharides, triterpenoids, alkaloids, nucleosides, and amino acids, exhibit various beneficial biological activities, including antioxidant, anticancer, and anti‐inflammatory effects. However, the medicinal properties of these bioactive compounds remain subject to controversy and ongoing challenges. This review outlines research on various active ingredients in *G. lucidum* and their anticancer effects, which may benefit immunocompromised individuals and biopharmaceutical developers, while providing fundamental knowledge on *G. lucidum* and its derived products.

## Graphical abstract

This paper systematically discusses various active compounds within *Ganoderma lucidum* and their application in the treatment of tumor diseases.

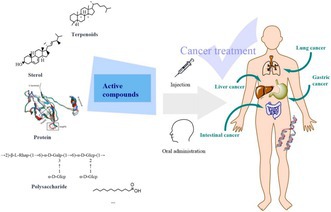

## 1 Introduction

Cancer is the most lethal disease in the world, and the human survival rate is limited due to its complex, diverse, and heterogeneous nature. Uncontrolled proliferation and inhibition of apoptosis are central to tumorigenesis initiation and progression. The balance between cell proliferation and apoptosis is crucial in determining the overall growth dynamics of a tumor, as well as its response to any therapeutic interventions or medications (Farheen et al. 2024). As such, current clinical treatment options for cancer are limited, and the pursuit of effective therapeutic interventions has been an arduous yet relentless endeavor (Robatel and Schenk 2022). Current therapeutic options for treating cancer mainly include surgery, radiation, chemical agents, biological agents, and cell therapies (Cravo and Mrsny 2020). These approaches have undoubtedly contributed to improving patient survival rates and quality of life in numerous instances; however, they are not devoid of substantial clinical side effects that pose substantial challenges in the holistic management of the disease (Burguin et al. 2021). Considering this, the exploration of novel, non‐toxic, stable, and reliable anticancer methods remains an urgent research topic.

Numerous natural products and their derivatives have blossomed into successful clinical antitumor medications, such as paclitaxel (Huang et al. 2025) and camptothecin (Liu and Yao 2025), offering hope to cancer patients. Among the myriad sources of these natural gifts, medicinal fungi have garnered considerable attention, thanks to their distinctive biological attributes and an array of diverse activities. *Ganoderma lucidum* is a fungus that can be used in both medicine and food (Sabarathinam et al. 2024). It is known by various names in different countries, such as Lingzhi (in China), Reishi, Youngzhi, and Linh Chi (Wu et al. 2024). As modern science and technology continue to advance, research into *G. lucidum* has evolved from traditional empirical practices into profound scientific endeavors. Numerous studies have revealed that *G. lucidum* contains many biologically active constituents, including polysaccharides, triterpenoids, nucleosides, sterols, alkaloids, amino acids, peptides, and trace elements, among others. These constituents form the basis for the medicinal properties of *G. lucidum* .

Among them, the polysaccharides and triterpenoids of *G. lucidum* are considered the most extensively researched active ingredients for their antitumor prowess (Plosca et al. 2025). The polysaccharides, with their highly branched structure and intricate connectivity, activate immune cells such as macrophages, natural killer (NK) cells, and T lymphocytes (Blundell and Camilleri 2022), which in turn stimulate cytokine secretion, enhancing immune surveillance and tumor cell eradication. Furthermore, they suppress tumor cell proliferation and induce apoptosis, among other effects (Zhong et al. 2022). Triterpenoids exhibit diverse varieties, encompassing ganoderic acids, alcohols, and ketones among hundreds of monomeric compounds (Zhao, Xu, and Zhong 2011). These compounds exhibit remarkable anti‐inflammatory, antitumorigenic, anti‐HIV, and hypolipidemic activity (Cör et al. 2018). They exert potent antitumor effects through mechanisms such as cell cycle regulation, induction of cancer cell apoptosis, and inhibition of tumor angiogenesis (Rahimnia et al. 2023).

In recent years, research into the antitumor efficacy of *G. lucidum* has made remarkable strides (Table 1). Spanning cellular experiments, animal models, and clinical studies, it has comprehensively revealed the antitumor activity and underlying mechanisms of *G. lucidum* and its bioactive constituents. In cellular experiments, these constituents directly inhibit the growth and proliferation of diverse tumor cells, including lung, liver, breast, colon, and rectal cancers, among others (Ye et al. 2023). Animal model studies further affirm that extracts of *G. lucidum* or its active components markedly hinder tumor progression and metastasis, thereby extending the survival of tumor‐bearing animals. Preliminary clinical studies have also validated the efficacy and safety of *G. lucidum* as an adjuvant in tumor treatment, enhancing the quality of life for cancer patients and mitigating the adverse effects of chemotherapy and radiotherapy.

**TABLE 1:** Representative examples of *G. lucidum* active compounds reported in the last 10 years.

Year	Category	Compound name	Name of researchers	Significant effects or means	References
2014	Polysaccharides	*G. lucidum* polysaccharide	Shen et al.	Direct inhibition of human hepatocarcinoma cells via liver gene regulation	Shen et al. (2014)
2015	Triterpenes	Ganoderenic acid B	Liu et al.	Enhanced cytotoxicity of chemotherapy drugs against ABCB1‐mediated multidrug‐resistant cancer cells	Liu et al. (2015)
	Polysaccharides	*G. lucidum* polysaccharide	Raj and Sa	Disruption of microtubule network in cancer cells that induces cell death	Raj and Sa (2015)
	Peptide	LZ‐8	Wu et al.	Suppression of hepatocellular carcinoma tumor progression by blocking c‐Met‐dependent or c‐Met‐independent pathways	Wu et al. (2015)
2016	Triterpenes	*Ganoderma* triterpenes	Shao et al.	Identification of 19 high‐frequency targets related to cancer and metabolic syndrome	Shao et al. (2016)
	Polysaccharides	Intracellular *G. lucidum* polysaccharide	Sui et al.	Demonstration of steady and prompt inhibitory effects on cancer cells	Sui et al. (2016)
2017	Polysaccharides	*Ganoderma* polysaccharides	Yu et al.	Enhancement of radiosensitivity in hepatocellular carcinoma cells via regulation of Akt signaling pathway	Yu et al. (2017)
	Triterpenes	Ganoderic acid A	Wang et al.	Induction of G0/G1 cell cycle arrest and apoptosis, and suppression of migration/invasion in human hepatocellular carcinoma cells	Wang et al. (2017)
	Peptide	LZ‐8	Lin et al.	Targeting of epidermal growth factor receptor overexpression/mutation and epidermal growth factor receptor‐dependent oncogenic processes	Lin et al. (2017)
2018	Polysaccharides	*G. lucidum* polysaccharide	Wu et al.	Induction of apoptosis in hepatocellular carcinoma cells	Wu et al. (2018)
	Triterpenes	Ganoderic acid A	Gill, Kumar, and Navgeet	Inhibition of proliferation, viability, and intracellular ROS levels in pancreatic cancer RIN‐5F cells by Ganoderic acid A (the best‐docked isoform) in a dose‐dependent manner	Gill et al. (2018)
2019	Polysaccharides	*Ganoderma* polysaccharides	Pan et al.	Inhibition of tumor growth and autophagy flux in vivo	Pan et al. (2019)
	Sterol	Ergosterol peroxide	Martínez‐Montemayor et al.	Induction of G1 phase cell cycle arrest, apoptosis via caspase 3/7 activation, and PARP 41 cleavage	Martínez‐Montemayor et al. (2019)
	Triterpenes	Ganoderic acid A	Cheng and Xie	Promotion of U251 growth inhibition, invasion/migration arrest, apoptosis, and autophagy via PI3K/AKT pathway inactivation in human glioblastoma	Cheng and Xie (2019)
2020	Polysaccharides	*G. lucidum* polysaccharides	Hsu et al.	Inhibition of activity and mobility of lung cancer cells	Hsu et al. (2020)
	Peptide	LingZhi oligopeptides	Liu et al.	Major apoptosis induction in A549 lung cancer cells via mitochondrial dysregulation	Liu, Yuan, et al. (2020)
2021	Triterpenes	Lucidadiol	Shin et al.	Induction of apoptosis and suppression of cell motility in B16 melanoma cells	Shin et al. (2021)
	Polysaccharides	*G. lucidum* spore polysaccharide	Song et al.	Alteration of macrophage polarity and potential reshaping of tumor microenvironment activity	Song et al. (2021)
2022	Polysaccharides	*G. lucidum* polysaccharides	de Camargo et al.	Alteration of cell morphology/granularity, delayed migration, reduced colony/sphere formation, and induction of non‐invasive, less proliferative tumoral cell behavior	de Camargo et al. (2022)
	Triterpenes	Lucidumol A	Shin et al.	Suppression of cell proliferation and migratory ability, and increased cell death	Shin et al. (2022)
2023	Polysaccharides	*G. lucidum* polysaccharide	Rahimnia et al.	Increase of prostate cancer cell sensitivity to drugs	Rahimnia et al. (2023)
	Triterpenes	Ganoderic acid A	Song et al.	Synergistic enhancement of oxaliplatin‐mediated tumor suppression, possibly via increased T cell cytotoxicity	Song et al. (2023)
2024	Polysaccharides	*G. lucidum* spore polysaccharide	Liu et al.	Major inhibition of tumor cell growth	Liu et al. (2024)
		*G. lucidum* polysaccharide	Li et al.	Major activation of T cell‐mediated antitumor immunity and enhanced anti‐PD‐1 immunotherapy efficacy in colorectal cancer	Li et al. (2024)
	Triterpenes	Ganoderic acid D	Luo et al.	Attenuation of gemcitabine resistance in triple‐negative breast cancer cells via inhibition of HIF‐1α‐dependent glycolysis	Luo et al. (2024)
2025	Polysaccharides	*G. lucidum* ‐extracted polysaccharides	Zangeneh et al.	Prevention of abnormal cell proliferation	Zangeneh et al. (2025)
	Functional polysaccharide	A fucogalactan of *Ganoderma* tsugae (GTP‐a2)	Zhang et al.	Alteration of cancer‐related metabolites, notably increased ophiobolin A level	Zhang et al. (2025)
	Triterpenes	Ganoderenic acid A and Ganoderenic acid B	Zhao et al.	Suppression of enhancer‐associated lncRNA FR121302 inhibits hepatocellular carcinoma growth	Zhao et al. (2025)

Despite notable research advancements in *G. lucidum*'s antitumor studies, numerous challenges and unresolved questions persist. The content and composition of active ingredients in *Ganoderma* exhibit substantial disparities across species, origins, and cultivation techniques, presenting formidable hurdles for quality control and standardization. These discrepancies further complicate clinical efficacy and safety assessments of *Ganoderma*‐derived therapies. Moreover, the anticancer mechanisms of *G. lucidum* still require further in‐depth investigation, including the interaction modes between its bioactive components and tumor cell surface receptors, as well as its metabolic pathways and pharmacokinetic characteristics in vivo. Such knowledge is critical for developing effective Ganoderma‐based antitumor drugs. Currently, clinical research on *G. lucidum* remains relatively scarce, with insufficient large‐scale clinical trials to comprehensively validate its therapeutic efficacy and safety. This evidence gap significantly limits its broader clinical application in cancer treatment.

Therefore, this review provides an in‐depth exploration of research on the anticancer compounds of *G. lucidum* , holding significant theoretical importance and practical value. Through systematic analysis of the bioactive anticancer components in *Ganoderma* and their action mechanisms, this work aims to enrich the theoretical framework of natural product‐based antitumor research. The findings may offer novel insights and potential drug candidates for developing new‐generation antitumor agents with enhanced efficacy and reduced toxicity. Moreover, this research may ultimately contribute to safer and more effective therapeutic options for cancer patients.

## 2 Main Bioactive Components

### 2.1 Polysaccharides

*Ganoderma* polysaccharides (GLPs) are among the most abundant components in *G. lucidum* , contributing to a major group of bioactive constituents (Hsu et al. 2017). GLPs comprise various monosaccharides (Gao and Homayoonfal 2023). Different GLPs are formed from complexes of homoglucans, hetero‐β‐glucans, heteroglycans, and α‐manno‐β‐glucan compositions (Gao and Homayoonfal 2023). Homoglucans have linear or branched structures with a backbone consisting of α‐ or β‐linked glucose monomers such as (1 → 6)‐β‐glucans and (1 → 6)‐α‐glucans. Furthermore, homoglucans have side chains attached to the polymer structures at different sites. Different side chains can be attached to the main chain of a homoglucan structure via various chemical bonds at different locations (Huang and Nie 2015). Heteroglucans such as arabinose, galactose, glucuronic acid, mannose, ribose, and xylose are the primary constituents of various compositions. In addition, GLPs have a complex, tertiary (triple helix) structure with a molecular weight distribution of 10^3^–10^6^ Da (Lu et al. 2020). The molecular weight (You et al. 2011), structural features, substituents, solubility, sugar type, and route of GLP administration substantially affect its bioactivity (Sun et al. 2012). The purpose of modifying GLPs is to graft specific functional groups that can regulate their solubility properties and provide desirable functionalities, thereby substantially improving their biological activity and antitumor properties (Luo et al. 2021). For instance, to enhance GLP water solubility, a common practice is to graft hydrophilic functional groups; for hydrophobic modification, substituting functional groups helps increase polysaccharide solubility in organic solvents while reducing it in water (Yu et al. 2019). GLPs are water‐soluble but insoluble in organic solvents. Therefore, they can be isolated by water extraction and alcohol‐induced precipitation, which generally produces low‐viscosity products (Lu et al. 2020). However, these products contain many impurities; therefore, column chromatography (Superdex‐200) and other methods can be used to purify GLPs by removing pigments, proteins, monosaccharides, and other contaminants (Chen et al. 2008). The hot water extraction method is easy to perform, safe, preserves polysaccharide integrity, and is suitable for industrial polysaccharide extraction. However, the hot water extraction method is time‐consuming and has a low yield. It can only extract the exopolysaccharides of *G. lucidum* with low biological activity.

Microbial fermentation offers a sustainable, green, efficient, and eco‐friendly method for extracting polysaccharides with significant industrial potential (Wang, Zheng, Zhou, et al. 2024). By carefully selecting the culture medium and optimizing fermentation conditions, high‐quality polysaccharides can be produced for various applications. This approach not only supports sustainable production but also aligns with the growing trend of using renewable resources and reducing environmental impact.

**FIGURE 1:**
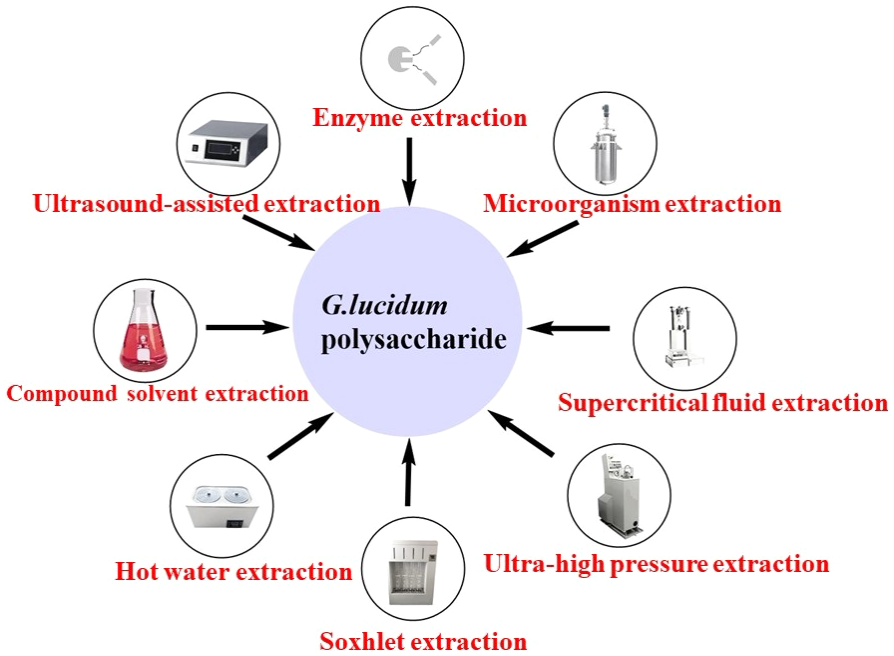
Methods used for the isolation of polysaccharides from *G. lucidum* .

To improve extraction efficiency and enhance the bioactivity of plant‐based polysaccharides, Wang, Zheng, Dai, et al. (2024) reviewed probiotic fermentation methods that substantially increase yield and bioactivity. This may be because polysaccharides can act as substrates for probiotic proliferation. Meanwhile, probiotics can degrade plant polysaccharides and produce bioactive substances that improve human health. Therefore, microbial fermentation enables polysaccharide extraction and production with improved nutrient bioavailability, sensory properties, and functional properties. This method is suitable for GLP extraction and production. However, in actual production, several methods are often combined to ensure greater extraction efficiency and production of highly active GLPs (Huang and Ning 2010) (Figure 1). GLPs can be used as an adjunct to anticancer drugs such as 5‐fluorouracil (Opattova et al. 2019), inulin (Liu et al. 2019), and doxorubicin (DOX) to increase their efficacy. GLPs enhance the cytotoxic or hypoglycemic effects of these drugs on malignant cells, reduce their toxicity to non‐malignant cells, and prevent reactive oxygen species (ROS) accumulation. Liang et al. (2014) assessed the inhibitory and apoptosis‐inducing effects of GLPs on human colon cancer (HCT‐116) cells in vitro. They observed that GLPs reduced the viability of HCT‐116 cells by programmed apoptosis in a time‐ and dose‐dependent manner. Furthermore, DNA fragmentation and decreased mitochondrial membrane potential were observed; Western blot analysis confirmed that GLPs upregulated Bax/Bcl‐2, caspase‐3, and poly(ADP‐ribose) polymerase (PARP) expressions. The results revealed that GLPs induce cytotoxicity and apoptosis in HCT‐116 cells. Raj and Sa (2015) observed that GLP yielded better results and specificity in HeLa cells. Nuclear staining revealed that HeLa and MCF‐7 cells exhibited severe damage, characterized by apoptotic morphological alterations, including nuclear and cytoplasmic condensation, reduced cell volume, and fragmentation. Fang et al. (2022) studied the extraction process and medicinal value of polysaccharides from *G. lucidum* spores. The results revealed that the extraction yield of the crude water‐soluble GLPs was 23.7% and the total carbohydrate content was 83.3%. Furthermore, it effectively inhibited HCT‐116 and NCI‐H460 xenograft tumor growth and tumor‐induced splenomegaly.

Recent research has focused on GLP receptors and downstream signal transduction pathways. Many studies have only briefly described which signaling molecules GLP action, whereas few have explored receptor and signal transduction mechanisms and systematically analyzed GLP regulatory effects on cell signaling pathways at the cellular, molecular, and genetic levels (Lu et al. 2020). It has been shown that complex signal transduction pathways are often interrelated via network regulation; however, more detailed and in‐depth studies are needed to explore these mechanisms (Yang et al. 2016).

### 2.2 Sterols

*G. lucidum* sterols (GLSs) are key secondary metabolites of *G. lucidum* and among the most important active components involved in inducing tumor cell death (Xu, Xiao, et al. 2021). GLSs are components of the *G. lucidum* cell membrane involved in regulating membrane fluidity, permeability, and membrane‐associated metabolism as byproducts of the isoprenoid biosynthesis pathway via squalene transport by acetyl‐CoA (Shahzad et al. 2017). *G. lucidum* contains numerous sterols, including lanosterols, ergosterols, and their derivatives, and approximately 27 GLS types have been identified to date (Wu et al. 2024). Li et al. (2016) purified ergosterol peroxide (5α,8α‐epidioxiergosta‐6,22‐dien‐3β‐ol), which has been reported to induce human cancer cell death, from *G. lucidum* and examined its biological functions. Various experiments have confirmed that ergosterol peroxide induces cell death and inhibits cell migration, potentially related to the expression of Foxo3 mRNA and protein in HepG2 cells. Ergosterol peroxide inhibits oncogenic AKT and c‐Myc, thereby activating Foxo3 expression, which in turn promotes downstream apoptosis‐related genes such as *PUMA* and *BAX*, initiating cancer cell apoptosis pathways. Xu, Xiao et al. (2021) isolated GLSs using a two‐phase aqueous system and studied their anti‐inflammatory mechanisms. They showed that the anti‐inflammatory GLS activity is mediated via the p38MAPK and NF‐κB signaling pathways. GLSs inhibit the p38MAPK pathway by blocking phosphorylation and also inhibit IκBa phosphorylation and degradation, thereby preventing NF‐κB p65 phosphorylation and inhibiting the NF‐κB signaling pathway. Chen et al. (2017) developed ergosterol peroxide from *G. lucidum* that could inhibit cancer growth by upregulating multiple tumor suppressors. Furthermore, they isolated 14 ergosterol derivatives with antitumor and antiangiogenic activities from *G. lucidum* and intensively investigated their structure–activity relationships for inhibiting HepG2 cells. They discovered that the highest ergosterol content is found in *G. lucidum* mycelia, providing a basis for further research and development of ergosterol derivatives. Wu et al. (2012) identified and purified ergosterol peroxide from *G. lucidum* and observed that it could overcome drug resistance conferred by miR‐378, further elucidating its mechanism in inducing miR‐378‐associated cancer cell death. Zheng et al. (2018) extracted 9,11‐dehydroergosterol peroxide (9[11]‐DHEP) from *G. lucidum* and used an MTT assay to investigate its antitumor effects and assess cell viability in various tumor and normal cell lines. The results revealed clear antitumor activity in several tumor types. Furthermore, they revealed that the antitumor effect may be attributed to decreased Mcl‐1 protein levels, mitochondrial membrane damage, cytochrome‐C release, and other mechanisms. Lee et al. (2009) extracted four steroids from the fruiting bodies of *G. lucidum* and investigated their cytotoxic activity against HL‐60, MCF‐7, and lung carcinoma cell lines. Moreover, *G. lucidum* extracts inhibited primary solid tumor growth in the spleen and prevented liver metastasis. Similarly, Lee, Hung, et al. (2011) studied the cytotoxic activities of steroids from the fruiting bodies of *G. lucidum* . Ergosta‐7,22‐diene‐2β,3α,9α‐triol was found to induce apoptosis in HL‐60 human promyelocytic leukemia cells by activating DNA fragmentation and caspase‐3 activity.

From the above examples, it can be concluded that sterols, as naturally occurring components in *G. lucidum*, possess considerable potential as chemopreventive and chemotherapeutic agents in cell culture studies and may help prevent various cancer types.

### 2.3 *G. lucidum* Triterpenoids

**FIGURE 2:**
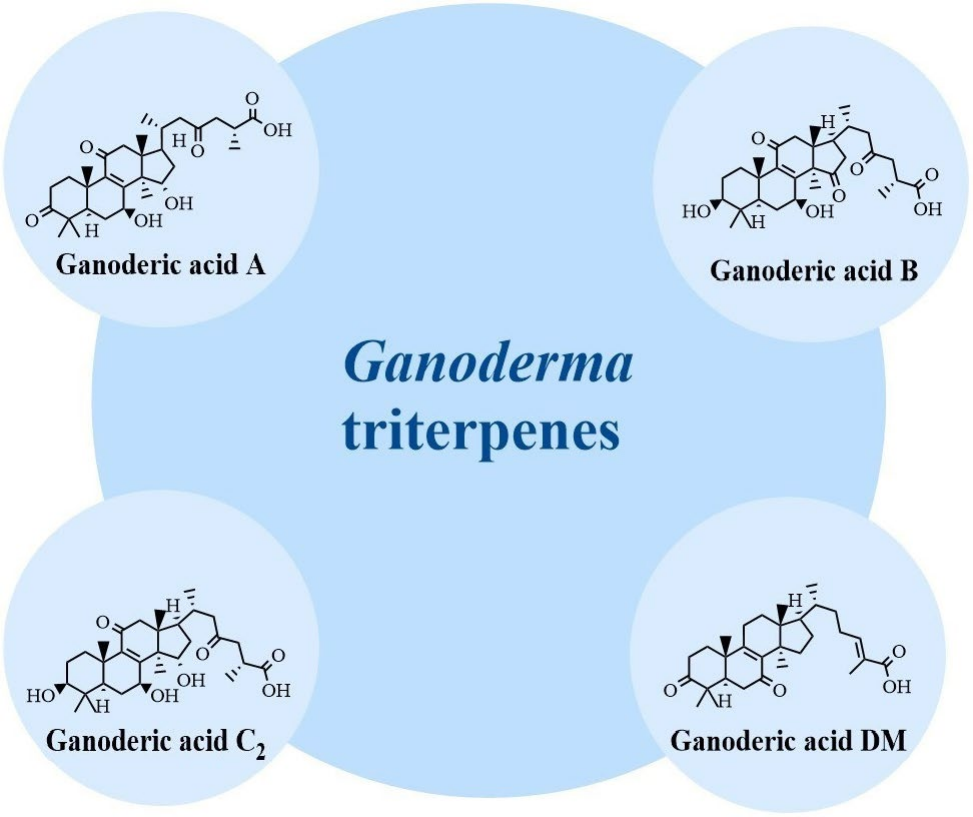
Chemical structures of triterpenoids in *G. lucidum* (Benkeblia 2015; Shao et al. 2016).

*G. lucidum* triterpenoids (GLTs) are bioactive components with important pharmacological properties (Lu et al. 2020; Xu, Chen, et al. 2021). GLTs are highly oxidized lanostane derivatives with high fat solubility, and different triterpene types and levels are found in the fruiting bodies and mycelia of *G. lucidum* (Xie et al. 2020). Most triterpenoids are tetracyclic or pentacyclic, while others are linear, monocyclic, bicyclic, and tricyclic (Figure 2). Tetracyclic triterpenes in *G. lucidum* are highly oxidized lanosterane‐type triterpenoid derivatives (Gao et al. 2018; Wang et al. 2020).

The fruiting bodies and mycelia of *G. lucidum* contain a diverse array of triterpenoids, varying in both type and concentration. The structural backbone of triterpenoids originates from lanosterol, featuring a tetracyclic structure with the molecular formula C_30_H_54_. Notably, most triterpenoids found in *G. lucidum* exhibit a high degree of oxidation. Differences in functional groups, including acids, alcohols, ketones, esters, and aldehydes, as well as variations in substituent positions, directly determine the medicinal properties of triterpenes (Wang et al. 2020). Currently, more than 380 triterpenoids have been isolated from the fruiting bodies, spores, and mycelia of *G. lucidum* , including ganoderic acid, ganoderic alcohol, and ganoderic spironolactone (Zheng et al. 2023). Most triterpenoid skeletons in *G. lucidum* consist of 27 or 30 carbon atoms, with only a minority featuring 24 carbon atoms (Xia et al. 2014). These variations primarily arise from differences in the substituent attached at the C‐17 position (Table 2).

**TABLE 2:** Selected triterpenoids and their bioactivities from *Ganoderma* (Galappaththi et al. 2022).

No.	Trivial name	Bioactivity (IC50/MIC or ED50)	Discoverer	Discovery year	Structure	References
1	Ganoderic acid A	Promising anticancer agent (via potent inhibitory effect)	Kubota et al.	1982	7β,15α‐ Dihydroxy‐3,11,23‐trioxo‐5α‐lanost‐8‐en‐26‐oic acid	Chen and Chen (2003), Kubota et al. (1982), Yao et al. (2012)
2	Ganoderic acid B	Moderately active inhibitor against HIV‐1 PR (0.17 mM)	Kubota et al.	1982	3β, 7β‐dihydroxy‐1 L, 15,23‐trioxo‐5α‐lanost‐8‐en‐26‐oic acid	Chen and Chen (2003), El‐Mekkawy et al. (1998), Kubota et al. (1982)
3	Ganoderic acid GS‐1	Anti‐HIV protease (58 μM)	Sato et al.	2009	7b hydroxy‐3,11,15‐trioxo‐lanosta‐8,24(E)‐dien‐26‐oic acid	Sato et al. (2009)
4	Ganoderic acid GS‐2	Anti‐HIV protease (30 μM)	Sato et al.	2009	7b,15a‐dihydroxy‐3,11‐dioxo‐lanosta‐8,24(E)‐dien 26‐oic acid	Sato et al. (2009)
5	Ganoderic acid GS‐3	Anti‐HIV protease—NE	Sato et al.	2009	12b‐acetoxy‐3b,7b‐dihydroxy‐11,15‐dioxo‐lanosta‐8,24(E)‐dien‐26‐oic acid	Sato et al. (2009)
6	Ganoderic acid Df	Humanaldose reductase inhibitory activity (22.8 M/mL)	Fatmawati, Shimizu, and Kondo	2010	7β, 11β‐dihydroxy‐3, 15, 23‐trioxo‐5α‐lanosta‐8‐en‐26‐oic acid by 1D‐ and 2D‐NMR spectra	Fatmawati et al. (2010)
7	Methyl ganoderate A acetonide	Anti‐AChE (18.35 μM), anti‐BChE—NE	Lee et al.	2011	methyl 7b,15a‐isopropylidenedioxy‐3,11,23‐trioxo‐5a‐lanost‐8‐en‐26‐oate	Lee, Ahn, et al. (2011)
8	Lucialdehyde E	Cytotoxic activity against esophageal tumor EC109 cell line (18.7 mg/mL)	Ma et al.	2012	7b,15a‐dihydroxyl‐3,11‐dioxo‐5a‐lanosta‐8,24‐dien‐26‐al	Ma et al. (2012)
9	Ganodecalone A	Cytotoxicity against K562 (17.22 μM)	Huang et al.	2017	(17R,20R,24E)‐26 hydroxy‐lanosta‐8,24‐dien‐3,11‐dione	Huang et al. (2017)
10	Lucidumol D	Selective antiproliferative and cytotoxic effects	Satria et al.	2019	(24S)‐11α,24,25‐trihydroxy‐5α‐lanost‐8‐ene‐3,7‐dione	Satria et al. (2019)

Triterpenoids have numerous biological effects, such as decreasing blood pressure, protecting the liver, lowering cholesterol levels, and exhibiting antitumor effects (Wang et al. 2020). Thyagarajan et al. (2010) demonstrated that GLTs suppress the proliferation of human colon cancer (HT‐29) cells and inhibit tumor growth. Furthermore, they demonstrated the anticancer effects of *G. lucidum* triterpenes after intraperitoneal GLT injection and concluded that GLTs exert systemic effects and inhibit the growth of HT‐29 cells. Zhang et al. (2022) isolated 10 lanostane triterpenes from statically cultured *G. lucidum* mycelia and observed that they had specific inhibitory effects on H_2_O_2_‐induced HepG2 cells. Moreover, they isolated 12 ganoderic acid derivatives and showed that they had notable liver protective effects. Kou et al. (2021) used immunofluorescence and molecular docking technology to confirm the inhibitory activity of ganoderterpene A and found that it significantly inhibited the MAPK and TLR‐4/NF‐κB signaling pathways and effectively improved mitochondrial membrane potential and GLP‐induced apoptosis. These results indicate that ganoderterpene A plays a protective role in microglial apoptosis by inhibiting inflammatory responses.

Many useful measures can be implemented to improve the extraction efficiency of GLTs; for example, in the follow‐up process, silica gel column chromatography or high‐performance liquid chromatography can be used to obtain more purified triterpenes (Lin and Yang 2019). Furthermore, the synthetic GLT production is also being actively explored, and the necessary enzyme genes involved in GLT biosynthesis have been discovered (Galappaththi et al. 2022). These enzymes are important in the triterpenoid synthesis pathway in *G. lucidum* ; thus, enzyme expression levels may directly or indirectly affect the structure and content of triterpenoids in *G. lucidum* . Furthermore, cytochrome P450 inducers, such as carbamazepine and phenobarbital, can induce GLT synthesis. Zhang et al. (2009) investigated the acute ganoderiol F and ganoderic acid A toxicity and found that the median lethal doses of ganoderiol F and ganoderic acid A were 2.95 mg/kg (with 95% reliability [2.59–3.40 mg/kg]) and 178.57 mg/kg (with 95% reliability [165.2–215.4 mg/kg]), respectively, suggesting that ganoderiol F is more toxic than ganoderic acid A. Therefore, 0.5 mg/kg doses of ganoderiol F and ganoderic acid A were administered intravenously in pharmacokinetic experiments. However, neither compound was notably toxic after oral administration at doses of 20–50 mg/kg. These preliminary experiments showed that, unlike ganoderic acid, ganoderiol F was fairly toxic to animals, especially after intravenous administration.

Maceration, a conventional method using organic solvents as extraction agents, is widely adopted for GLT extraction (Zheng, Zhu, et al. 2020). Novel microwave irradiation or ultrasound‐assisted methods have been effectively used to extract GLTs from *G. lucidum* (Chen et al. 2007). Currently, organic solvents such as chloroform and methanol are commonly used to extract GLTs at appropriate temperatures due to their ease of use; however, they present limitations such as long extraction time, low extraction efficiency, and residue formation (Ruan et al. 2014). In contrast, the supercritical fluid extraction method offers advantages such as low energy consumption, minimal degradation of active ingredients, and no residual organic solvent; thus, the GLT extraction rate is higher than with traditional methods. Ji et al. (2011) extracted triterpenes from the fruiting bodies of *G. lucidum* using sequential hot water extraction followed by ethanol‐insoluble polysaccharide removal and gel‐filtration chromatography. They subsequently used the Alamar Blue assay, flow cytometry, and caspase‐3 activity assays to detect cell proliferation, apoptosis, and cellular activity. They observed that the triterpenes inhibited the growth of various tumor cells and significantly enhanced apoptosis in a dose‐dependent manner.

### 2.4 *G. lucidum* Alkaloids

**FIGURE 3:**
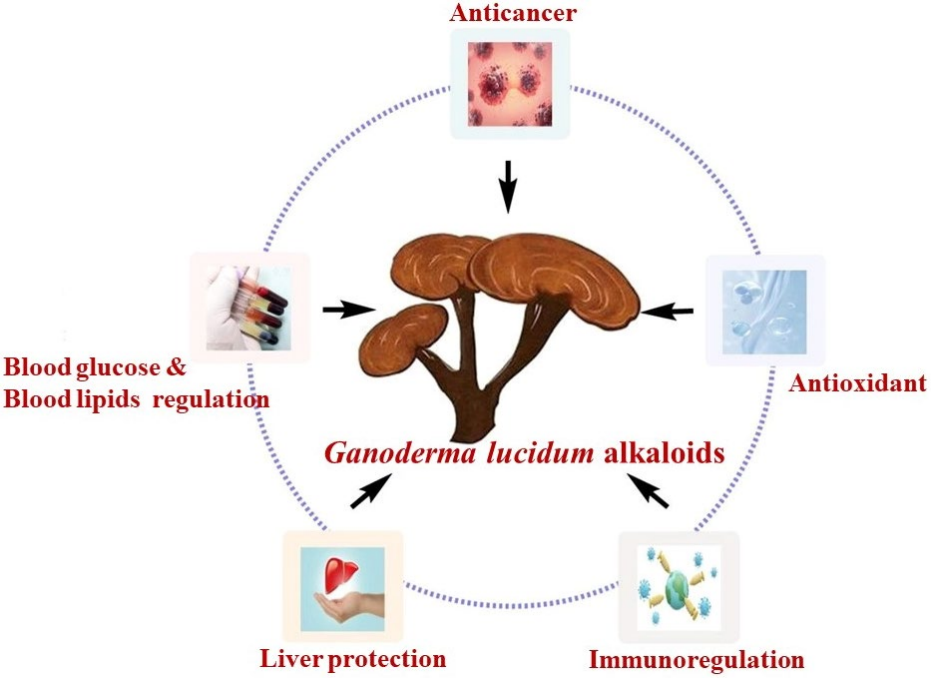
Alkaloids and their pharmacological significance.

Alkaloids, a class of alkaline organic compounds, are among the most important secondary metabolites in *G. lucidum* (Sharma, Bhardwaj, et al. 2019). Alkaloids are primarily synthesized from various amino acids or their derivatives; they are structurally complex and contain cyclic ring structures with one or more basic nitrogen atoms (Olofinsan et al. 2023). In particular, alkaloids contain heterocyclic compounds that substantially enhance their biological activities (Dai et al. 2018). Various alkaloids have been identified in *G. lucidum* ; given their structural diversity, they vary in chemical and biological properties. In addition, alkaloids with similar ring structures but synthesized through different metabolic pathways may exhibit distinct pharmacological activities (Dey et al. 2020) (Figure 3). Alkaloids are insoluble in water and react with acids to form salts. Moreover, most alkaloids have a bitter taste, and their free bases are soluble in non‐polar organic solvents (Pereira et al. 2023).

Numerous methods have been used to extract alkaloids, including maceration, ultrasonic extraction, Soxhlet extraction, and accelerated solvent extraction (Wang et al. 2015). Reflux extraction, Soxhlet extraction, and maceration at room temperature are the most commonly used methods for alkaloid extraction. Ionic liquid and microwave‐assisted extraction are sometimes combined to improve the extraction efficiency of alkaloids (Teng and Choi 2014). For example, gradient concentration extraction using butanol or octanol from aqueous solution is a classic method for obtaining *G. lucidum* alkaloids (Gregorová et al. 2010). High‐throughput screening and computational methods can also be used to improve alkaloid extraction rates (Yan et al. 2021).

The alkaloids isolated from *G. lucidum* primarily include choline, betaine, *G. lucidum* base A, and γ‐trimethylaminobutyric acid. Zhang et al. (2021) isolated three mercaptoterpenoids and five alkaloids from the fruiting bodies of *G. lucidum* and reported substantial neuroprotective properties against glutamate‐induced excitotoxicity in SH‐SY5Y cells. Lu et al. (2019) isolated 12 aromatic compounds from *G. lucidum* , including four key alkaloids, and evaluated ganocochlearine A activity in relation to cell damage. They observed that ganocochlearine A had significant neuroprotective and anti‐inflammatory effects. Chen and Lan (2018) synthesized lucidimine B and C, and reported that lucidimine B had stronger antioxidant effects than its congener C, and exhibited antiproliferative properties in MCF‐7 cells. The chemical names, structural formulas, references, and information on other types of ingredients are listed in Table 3.

**TABLE 3:** Several alkaloids commonly found in *G.* ***lucidum*** (Wu et al. 2024).

Name	Molecular formula	Molecular weight (g/mol)	Source location	Discoverer	Discovery year	References
Ganoines I	C_11_H_17_NO_2_	195	Hypha of deep fermented *Ganoderma capense*	Yang and Yu	1990	Yang and Yu (1990)
Ganoines II	C_14_H_17_NO_2_	231	Hypha of deep fermented *G. capense*	Yang and Yu	1990	Yang and Yu (1990)
Sinensine	C_15_H_15_NO_3_	257	Fruiting bodies of *Ganoderma sinense*	Liu, Zhao, and Chen	2010	Liu et al. (2010)
Sinensine B	C_14_H_14_NO_2_	228	Fruiting bodies of *G. sinense*	Liu et al.	2011	Liu et al. (2011)
Sinensine C	C_14_H_14_NO_3_	244	Fruiting bodies of *G. sinense*	Liu et al.	2011	Liu et al. (2011)
Sinensine D	C_14_H_11_NO_3_Na	264	Fruiting bodies of *G. sinense*	Liu et al.	2011	Liu et al. (2011)
Sinensine E	C_15_H_14_NO_3_	256	Fruiting bodies of *G. sinense*	Liu et al.	2011	Liu et al. (2011)
Lucidimine A	C_16_H_16_NO_3_	270	Fruiting bodies of *Ganoderma lucidum*	Zhao et al.	2015	Zhao et al. (2015)
Lucidimine B	C_15_H_13_NO_2_	239	Fruiting bodies of *G*. *lucidum*	Zhao et al.	2015	Zhao et al. (2015)
Lucidimine C	C_16_H_16_NO_3_	270	Fruiting bodies of *G*. *lucidum*	Zhao et al.	2015	Zhao et al. (2015)
Lucidimine D	C_17_H_18_NO_4_	300	Fruiting bodies of *G*. *lucidum*	Zhao et al.	2015	Zhao et al. (2015)
Ganocalicine A	C16H15NO3	269	Fruiting bodies of *Ganoderma calidophilum*	Huang et al.	2016	Huang et al. (2016)
Ganocalicine B	C15H13NO2	239	Fruiting bodies of *G. calidophilum*	Huang et al.	2016	Huang et al. (2016)
Australine	C_14_H_13_NO_4_	259	Fruiting bodies of *Ganoderma australe*	Zhang et al.	2021	Zhang et al. (2021)

### 2.5 Others

The proteins present in *G. lucidum* , particularly fungal immunomodulatory proteins and glycoproteins, exhibit profound immunomodulatory effects and potent antitumor activity. These proteins activate the immune system, enhance immune cell activity, and suppress tumor growth and metastasis by strengthening the body's immune defenses. To date, proteins serving as primary immune functional components have been relatively understudied, and little research has compared the immune‐active proteins found in various mushroom species (Xu et al. 2023). However, advances in the study of diverse bioactive proteins in medicinal fungi, particularly fungal immunomodulatory protein discovery, have opened novel avenues for exploring their pharmacological mechanisms. A typical example is the Rho GTPase Rac, which belongs to a class of migration‐associated proteins that play a central role in cancer progression. As a key member of the Rho GTPase family, Rac is often found to be hyperactivated rather than mutated. This hyperactivation results from deregulation in the expression or activity of upstream regulators known as guanine nucleotide exchange factors (GEFs). Furthermore, mutations and impairments in the proteasomal degradation of Rac in tumors contribute significantly to its heightened activation state (Acevedo‐Díaz et al. 2019). Wertheimer et al. (2012) conducted a comprehensive review of Rac GTPases, a small G‐protein intricately involved in tumorigenesis and metastasis. These proteins serve as critical transducers of signals from tyrosine kinases, G‐protein‐coupled receptors, and integrins. They also tightly regulate several essential cellular functions, including motility, adhesion, and proliferation. Zheng, Zhang, and Liu (2020) identified anticancer peptides (ACPs) from *G. sinense* using proteomics coupled with advanced genomic data mining techniques. The results showed that 15 trypsin‐digested fragments from *G. sinense* proteins may serve as ACPs, with three matching known anticancer peptide sequences. However, the researchers only identified a method and did not isolate a pure sample of the anticancer peptide. Furthermore, Zhao et al. (2023) reviewed that GLAP, a protein from *G. lucidum* , can induce apoptosis in A549 lung tumor cells. It also inhibits the growth of human glioma U251 cells by inducing cell cycle arrest and promoting cell death. Additionally, it may exert anticancer effects by impeding tumor angiogenesis, thereby suppressing cancer development and spread.

Amino acids are the basic building blocks contributing to the chemical versatility of proteins and also serve as crucial sources for cellular metabolism and energy supply. Certain amino acids found in *G. lucidum* can inhibit tumor cell growth and induce apoptosis (Bojarska et al. 2019). For instance, arginine and glutamine are precursors for the biomass required for rapid cancer cell proliferation. They participate in tumor cell metabolism, disrupting energy supply and material synthesis, thereby inhibiting growth (Byun et al. 2020; Chen et al. 2021). Furthermore, amino acids in the fungus, such as leucine, can induce caspase‐dependent apoptotic death in cancer cells (Sheen et al. 2011). Hydrophobic amino acids, including phenylalanine, alanine, and proline, may also enhance free radical scavenging activity within peptides, thereby inhibiting cancer growth (Girjal et al. 2012).

Phenolic compounds are also an important class of antioxidants in *G. lucidum* . They can scavenge free radicals and reduce oxidative stress, thereby contributing to antioxidant and anticancer effects. Kolniak‐Ostek et al. (2022) used high‐performance liquid chromatography (HPLC) to identify 28 polyphenols from the fruiting bodies of *G. lucidum* . However, the phenolic acid content was relatively low, at only 912.38 mg per 100 g dry weight. These compounds showed strong antiproliferative effects on breast cancer cell lines (MCF‐7, MCF‐7/DX, MDA‐MB‐231) and intestinal cancer lines (LOVO, SW 620). This discovery underscores the potential role of *Ganoderma* extracts in combating oxidative stress‐related diseases, including cancer.

## 3 Mechanism of the Antitumor Action of *G. lucidum*

*G. lucidum* exhibits inhibitory effects on various malignant tumors, including liver, breast, lung, and colon cancers (Bu et al. 2024). Jiao et al. (2020) investigated the impact of GLSO on breast cancer cells, focusing on the molecular mechanisms underlying its anticancer properties. Their findings showed that the GLSO potently inhibited the proliferation of MDA‐MB‐231 cancer cells in vitro and effectively suppressed 4T1 tumor growth in vivo by modulating key genes and proteins involved in apoptosis. In vitro experiments demonstrated that GLSO substantially upregulated Bax and caspase‐3 expressions in MDA‐MB‐231 cells, with no discernible effect on caspase‐8 expression. Furthermore, the GLSO‐treated group exhibited a marked reduction in tumor growth in vivo. Western blot analysis corroborated the in vitro findings, reinforcing the observed effects. Notably, MDA‐MB‐231 cell co‐treatment with caspase inhibitors diminished the GLSO inhibitory effect on cell growth. GLSO exhibits antitumor activity by inducing apoptosis in MDA‐MB‐231 cells and tumors in vivo, likely via the mitochondrial apoptotic pathway. Kim et al. (2016) combined extracts from *G. lucidum* and *Polyporus umbellatus* and demonstrated their antiproliferative effects against malignant cells. They subsequently used annexin V‐propidium iodide staining and flow cytometry to measure cell proliferation. They observed that the mixture increased intracellular calcium concentration, induced ROS production, inhibited cell proliferation, and promoted apoptosis in MCF‐7 cells. Thus, different *G. lucidum* extracts and extraction methods may exert distinct antitumor effects. Numerous such examples exist, but their anticancer effects are primarily mediated through the following mechanisms.

### 3.1 Inhibition of Tumor Cell Proliferation and Migration

Antiproliferative and pro‐apoptotic agents are effective for tumor treatment, as tumor cell invasion and migration are major causes of cancer‐related death (Min et al. 2011). Ding et al. (2023) studied the effects of GLT on hepatocellular carcinoma (HCC SMMC‐7721) cells and in a nude mouse model. The results revealed that triterpenes inhibited tumor cell growth by suppressing proliferation, inducing apoptosis, and preventing migration and invasion, without causing notable toxicity to normal cells and tissues. Zhang (2017) investigated the effect of *G. lucidum* on the Wnt/β‐catenin signaling pathway and elucidated the molecular mechanism of its inhibitory effect on breast cancer cells. They observed that *G. lucidum* blocked the Wnt/β‐catenin signaling pathway by inhibiting the phosphorylation of the Wnt coreceptor LRP6, which, in turn, suppressed the growth and migration of breast cancer cells. Jiao et al. (2020) investigated the effect of *G. lucidum* spore oil on breast cancer cells and explored the potential molecular mechanisms underlying its anticancer activity. They observed that *G. lucidum* spore oil effectively inhibited MDA‐MB‐231 cancer cell proliferation in vitro and 4T1 tumor growth in vivo by regulating key genes and proteins involved in apoptosis. These results indicate that *G. lucidum* inhibits the proliferation and migration of tumor cells. Furthermore, some studies have reported that *G. lucidum* extracts inhibit tumor cell proliferation by regulating the cell cycle. For example, Cheng et al. (2020) used brain tumor cells and employed flow cytometry and western blotting to analyze cell viability. They investigated the effects of different *G. lucidum* extract concentrations on glioblastoma (GBM8901 and U87) cell survival rates after 24, 48, and 72 h of incubation. The survival rates of both cell lines were significantly reduced in a time‐ and dose‐dependent manner. After incubation with 10% *G. lucidum* extract for 24 h, the survival rate of GBM8901 and U87 cells decreased to 59% and 74%, respectively. The inhibitory effect was further enhanced after treatment for 48 and 72 h, indicating that long‐term treatment with *G. lucidum* may increase its inhibitory effect on mitochondrial metabolic activity in GBM8901 and U87 cells. Therefore, it was concluded that *G. lucidum* extract may induce apoptosis and inhibit glioblastoma cell migration. Li et al. (2019) purified and chromatographically fractionated the extract of white *G. lucidum* based on biological activity, enriched active components, isolated ganoderiol F, and treated MDA‐MB‐231 cells for 48 h. They reported promising results, showing that at a concentration of 44 μM, the number of cells in the G_0_/G_1_ phase increased by 17.5%, and those in the S and G_2_/M phases decreased by 15.0% and 2%, respectively. Compared with the control group, the G_1_ phase of the MDA‐MB‐231 cell cycle was significantly inhibited, while the S and G_2_/M phases were decreased. Therefore, ganoderiol F induces cell death by inhibiting cell proliferation in the G_1_‐S phase of the cell cycle. Through an in‐depth analysis, they speculated that ganoderiol F may downregulate the expression of cyclin‐related proteins, such as cyclin D1, CDK4, CDK6, cyclin E, and CDK2, thereby inhibiting cancer cell cycle progression.

### 3.2 Immunoregulation

Some active substances in *G. lucidum* have immunomodulatory effects, such as improving humoral immunity and enhancing cellular immunity, and a promotive effect on non‐specific immunity has also been reported. In addition, *G. lucidum* inhibits excessive immune responses and reduces autoimmune disease occurrence. Among the active compounds, GLPs are the most important and play important regulatory roles in vivo and in vitro (Lin 2005).

**FIGURE 4:**
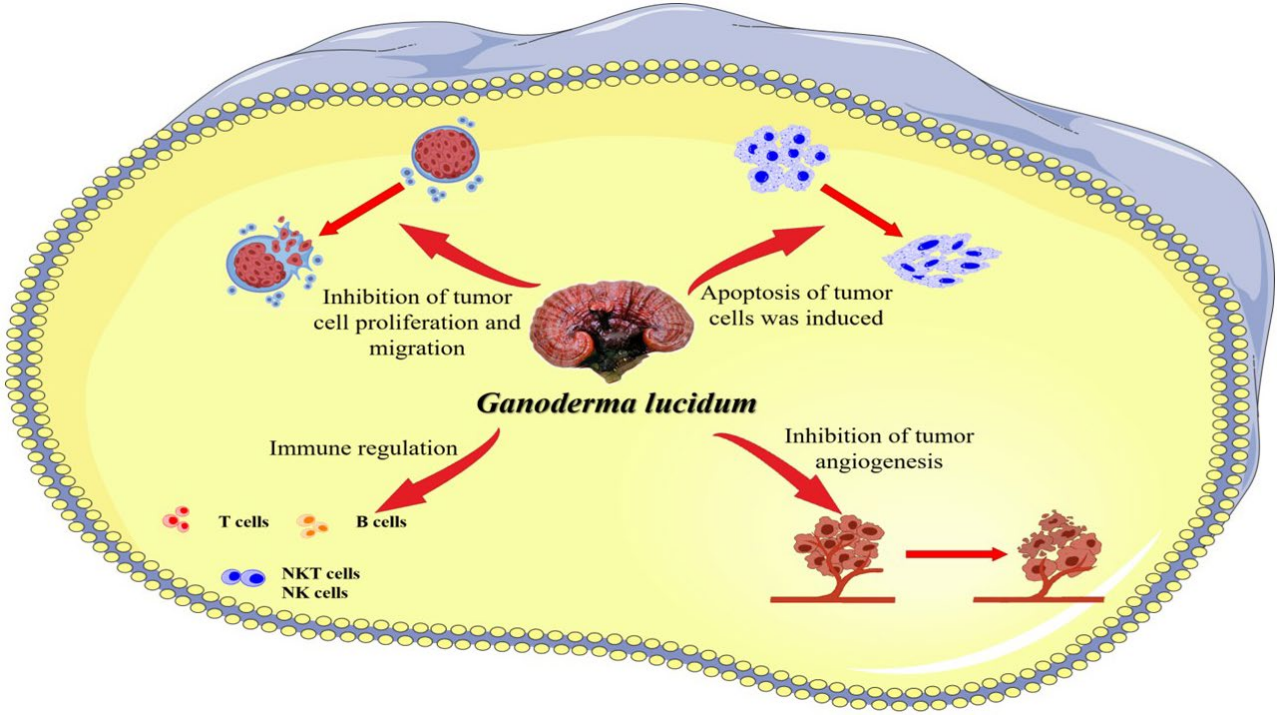
Molecular GLP mechanism in modulating immune effector cell activity.

The immune regulation mechanism by GLPs mainly involves improving the phagocytic ability of macrophages, promoting the proliferation and differentiation of immune cells, and enhancing the functions of NK cells, macrophages, lymphocytes, and dendritic cells (DCs). GLPs also promote immune cytokine production and improve immune function (Zhang et al. 2002). Macrophages phagocytose tumor cells, and antigen presentation plays an important role in inducing an antitumor adaptive immune response. Furthermore, GLPs can inhibit cancer cell growth through immune regulation, likely through macrophage polarization (Figure 4). Song et al. (2021) revealed a specific biological mechanism of *G. lucidum* spore polysaccharides in enhancing immunity and inhibiting liver cancer (H22) cell growth through experiments. These GLPs promoted primary macrophage polarization by activating them to reshape the tumor microenvironment. In addition, the GLPs promoted cytokine secretion, such as TNF‐α, IL‐1β, IL‐6, and TGF‐β1, as well as various inflammatory factors, and interacted with key genes and proteins to induce HCC cell apoptosis by regulating the PI3K/AKT metabolic pathway.

NF‐κB serves as a pivotal transcription factor, with its signaling pathway playing an essential role in the induction of numerous inflammation‐related cytokines and mediators, such as iNOS and TNF‐α, which are instrumental in modulating inflammatory responses and tumorigenesis. Based on this activity, Li, Tang, et al. (2023) proposed leveraging GLPs to activate MAPK/NF‐κB signal transduction pathways to regulate macrophage polarization and inhibit HCC cell proliferation. Their experiments revealed that GLP administration at doses of 100 and 200 mg/kg in mice increased p‐MEK and p‐ERK activity in macrophages, changed the phenotype of macrophages, and polarized macrophages, thereby effectively inhibiting tumor growth. Moreover, CD86 expression was higher in Hepa1‐6 allograft tumors in mice treated with GLPs at a dose of 200 mg/kg.

In T cell‐mediated antitumor immunity, Li et al. (2024) reported that GLPs significantly inhibited tumor growth in colorectal cancer and activated antitumor immunity. In the spleen and tumor tissues, the proportion of cytotoxic CD8 T cells and Th1 cells increased, whereas that of immunosuppressive regulatory T cells decreased. In addition, GLPs improved microbiome dysregulation and increased short‐chain fatty acid production, while reducing the kynuridine to tryptophan (Kyn/Trp) ratio in the serum, contributing to the antitumor immunity of T cells. Lo et al. (2024) discovered that a polysaccharide from *G. lucidum* (WSG) inhibits melanoma growth in mice carrying B16F10 cells. WSG downregulates the EMT‐associated transcription factors Snail and Twist and reduces protein levels in transforming growth factor β receptors (TGFβRs), thereby inhibiting the phosphorylation of intracellular signaling molecules, such as FAK, ERK1/2, and Smad2, which, in turn, inhibits cytotoxicity and melanoma cell movement.

In recent years, several studies have investigated proteins isolated from *G. lucidum* . Kino et al. (1989) isolated a new protein, LZ‐8, from *G. lucidum* , which exhibited mitotic activity in vitro and immunomodulatory activity in vivo. This protein promoted mitogen activity, induced T cell proliferation and intercellular aggregation, and substantially increased leukocyte adhesion molecule‐1 expression (ICAM‐1) (Haak‐Frendscho et al. 1993). Liang et al. (2012) studied a recombinant LZ‐8 (rLZ‐8) protein and found that it inhibited growth and induced cell death in human gastric cancer (SGC‐7901) cells. rLZ‐8 induced autophagic cell death by aggregating in the endoplasmic reticulum (ER), triggering ER stress and the ATF4‐CHOP pathway, and activating ubiquitin/proteasome ER‐related degradation systems. rLZ‐8 induced G1‐phase nuclear accumulation and S‐phase nuclear reduction; the autophagy arm of the system was overstimulated by excess rLZ‐8, leading to cell death via an autophagic response. rLZ‐8 was also used as a novel adjuvant to enhance DNA vaccine efficacy by activating DCs and promoting the innate and adaptive immune responses through TLR4, thereby improving therapeutic effects in tumors. Lin et al. (2011) demonstrated that rLZ‐8 activated mouse DCs through TLR4, which enhanced the therapeutic effect of the HER‐2/tumor in vivo.

*G. lucidum* can also inhibit the expression of programmed cell death protein 1 (PD‐1), which plays a key immunomodulatory role in tumor cells. As an immune cell surface protein, it interacts with programmed death ligand 1 (PD‐L1) to inhibit T cell proliferation and prevent autoimmune diseases; however, it also prevents the immune system from killing tumor cells, which can lead to cancer development. Therefore, the reduction in PD‐1 expression is an important mechanism of *G. lucidum* ‐mediated immunomodulation. Su et al. (2018) treated a T1‐breast cancer xenograft mouse model with an extract from *G. lucidum* (ESG) spores. ESG significantly reduced PD‐1 expression in the spleen and CTLA‐4 expression in tumor cells.

### 3.3 Tumor Cell Metabolism and Apoptosis

Apoptosis is a genetically controlled, orderly process of cellular self‐regulation driven by internal and external signal activation. Tumor cells escape apoptosis through various mechanisms, and the intrinsic apoptotic pathway in cancer cells is often disrupted and closely regulated by cellular metabolism (Sharma, Boise, and Shanmugam 2019). A reduction in anti‐apoptotic protein expression levels and pro‐apoptotic protein activation is crucial for the induction of apoptosis in multiple cancer cells (Peng et al. 2024). Zhao, Xu, and Zhong (2011) confirmed that *G. lucidum* induces apoptosis in tumor cells through the Akt signaling pathway and cell cycle G2/M phase arrest. Jin et al. (2020) reported that GLPs reduce activity and aggressiveness, block the cell cycle, and promote apoptosis in cervical cancer cells by inhibiting the epithelial‐mesenchymal transition and JAK/STAT5 pathways. At a dose of 200 μg/mL for 48 h, GLPs weakened the invasion and migration ability of cervical cancer cells, promoted apoptosis, and limited their cell cycle progression. Phosphorylated JAK and STAT5 expression levels were also reduced in GLP‐treated cervical cancer cells, indicating JAK/STAT5 signaling pathway inhibition. OuYang et al. (2013) demonstrated that GLPs induce cell cycle arrest in human hepatoma (HepG2 and Bel‐7404) cells by upregulating cyclin‐dependent kinase inhibitors and downregulating cyclins, thereby inducing apoptosis in both cell types via the AKT‐related mitochondrial apoptosis pathway.

Hsin et al. (2018) cloned and purified a recombinant fungal immunomodulatory protein from *Ganoderma microsporum* that induces lung cancer cell death by activating autophagy without inducing apoptosis. When human lung adenocarcinoma (A549) cells were injected into nude mice, oral *G. microsporum* administration significantly inhibited tumor growth and induced autophagy, revealing a novel role of *G. microsporum* in autophagy activation. Bai et al. (2020) evaluated the apoptotic effects of enzymatically hydrolyzed GLPs in human colon cancer (HTC‐116) cells using flow cytometry. The results revealed that EGLP may induce apoptosis in HCT‐116 cells by upregulating the expression of Bax, phosphorylated ERK, and cleaved caspase‐3, and by downregulating the expression of BCL‐2, phosphorylated Akt, and epoxide hydrolase.

Mitochondrial DNA elimination can limit tumor development; therefore, mitochondria play a multifunctional role in the progression of malignant tumors, making them a feasible therapeutic target. Liu et al. (2009) investigated whether *G. lucidum* and its extracts affect tumor cell metabolism and apoptosis via the mitochondrial transmembrane depolarization pathway and cell cycle arrest. They observed that the AKT signaling pathway indirectly participates in the intrinsic apoptotic pathway. Akt dephosphorylation enables the interaction between pro‐apoptotic protein Bax and the voltage‐gated anion channel in the mitochondrial outer membrane, which opens the mitochondrial permeability transition pore, promoting the release of mitochondrial intermembrane proteins and inducing apoptosis. Liu, Yuan, et al. (2020) explored the antitumor efficacy of a *G. lucidum* oligopeptide, LZO‐3, and concluded that it induces apoptosis via cell cycle arrest and mitochondrial dysfunction. In human lung tumor (A549) cells treated with LZO‐3 for 60 h, significant mitochondrial membrane potential disruption was observed, leading to the release of apoptotic factors. Simultaneously, the proportion of tumor cells in the G0 and G1 phases increased by 7.51% and 12.1%, respectively, thereby blocking cell cycle progression and ultimately inducing apoptosis. Liu et al. (2023) isolated the triterpenoid compound lucialdehyde B from *G. lucidum* and demonstrated that it effectively inhibits nasopharyngeal carcinoma (CNE2) cell proliferation and induces mitochondria‐dependent apoptosis. After treating CNE2 cells with a specific dose of lucialdehyde B for 48 h, the proportion of cells in the G2/M phase increased by 3.61%–9.15%, indicating that lucialdehyde B induced cell cycle arrest at the G2/M phase. Western blotting and fluorescence microscopy further confirmed that lucialdehyde B induces apoptosis in CNE2 cells via the mitochondrial pathway.

### 3.4 Inhibition of Tumor Angiogenesis

The formation of new blood vessels within a tumor is a key factor in tumor development. Tumor cells promote capillary growth into the tumor by secreting various growth factors, which provide essential nutrients, thereby promoting tumor expansion and metastasis. Therefore, inhibiting tumor angiogenesis is an important cancer treatment (Kao et al. 2013). The active compounds of *G. lucidum* can effectively inhibit angiogenesis, prevent vascular endothelial cell proliferation and migration, and suppress tumor protein expression or activity involved in angiogenesis (Lu et al. 2020). Vascular endothelial growth factor (VEGF) is an important regulatory factor for vascular endothelial cell function during tumor growth (Kleespies et al. 2004). Angiogenesis requires a certain number of endothelial cells to promote blood vessel growth; thus, vascular endothelial cells play a key role in this process. Inhibiting vessel generation is an effective antitumor pathway that prevents the functioning of tumor blood vessels. Dai et al. (2014) examined the effects of Cx43 knockdown on VEGF expression and cell proliferation in human ovarian cancer (HO 8910 [HOCC]) cells treated with *G. lucidum* . After 3 days, *G. lucidum* significantly increased Cx43 in the control group, inhibited VEGF expression in HOCC cells in a dose‐dependent manner, and decreased cell proliferation, indicating that Cx43 may have diagnostic value in ovarian cancer. Weng and Yen (2010) reported that the inhibitory effect of *G. lucidum* extract on metastatic prostate cancer (PC‐3) cells may be mediated by ERK1/2 and Akt kinases phosphorylation via extracellular signaling. The activity of activator protein‐1 (AP‐1) and nuclear factor‐kappa B (NF‐kB) was inhibited, and the secretion of VEGF and transforming growth factor‐β1 (TGFβ1) was reduced. Hsu et al. (2009) studied the antiangiogenic effect of *G. lucidum* methanol extract (GTME) on human epidermoid carcinoma (A‐431) cells. The results showed that GTME reduced VEGF levels to 1.5 mg/mL and inhibited the expression of epidermal growth factor receptor and VEGF in vitro and in vivo, ultimately suppressing capillary formation in human umbilical vein endothelial cells. Liu, Wang, et al. (2020) used GLTs for cancer treatment and achieved a tumor inhibition rate of 51.54%. Furthermore, mRNA and protein expression of VEGF receptors were reduced, indicating that GLTs play an important role in inhibiting tumor angiogenesis. In addition, *G. lucidum* can regulate apoptosis in vascular endothelial cells, thereby inhibiting angiogenesis. Cao and Lin (2006) investigated the effect of whole cell *G. lucidum* polysaccharide peptide extracts on lung cancer. The results revealed that a 100 μg/mL dose of this compound substantially decreased Bcl‐2 expression, increased pro‐apoptotic protein Bax expression, and induced apoptosis in cancer epithelial cells, which may explain the inhibition of tumor endothelial cell proliferation.

### 3.5 Reducing the Drug Resistance of Tumor Cells

**FIGURE 5:**
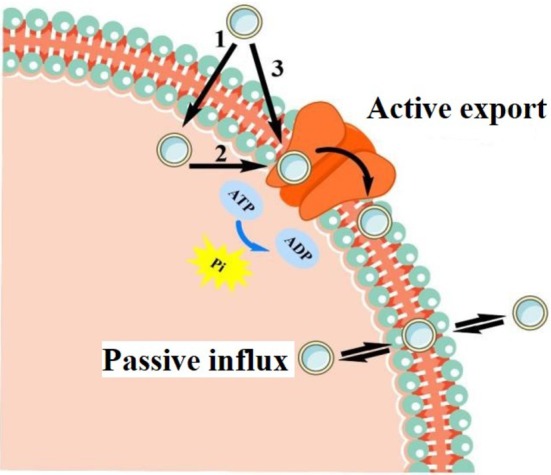
Schematic diagram of P‐glycoprotein‐mediated drug efflux.

Cancer treatments include chemotherapy, immunotherapy, and targeted therapy; however, current clinical treatments mainly rely on chemotherapy drugs (Anand et al. 2023). Although tumor therapy has entered the era of targeted therapy, targeted drugs can extend the survival of patients with advanced cancer for a limited period; however, most eventually develop drug resistance (De Conti et al. 2021). There are two primary mechanisms underlying drug resistance in cancer cells. First, resistance to a broad range of anticancer drugs is often acquired through the expression of one or more energy‐dependent transporters that regulate drug efflux from cells (Gottesman 2002). Second, acquired drug resistance predominantly results from molecular alterations in drug targets. Furthermore, epigenetic changes and the expression of drug resistance genes also affect the development of acquired drug resistance. Additionally, other mechanisms, such as secondary cell‐type transformation, induction of drug‐detoxifying pathways, and activation of bypass mechanisms, occur within exosomes (Li et al. 2022). Abnormal metabolic activity of cancer cells plays a crucial role in suppressing antitumor immune response, thereby facilitating tumor immune evasion and metastasis (Martinez‐Outschoorn et al. 2017). The tumor microenvironment (Quail et al. 2016) and non‐coding RNAs (ncRNAs), such as circRNAs, lncRNAs, and miRNAs (Calin and Croce 2006), are involved in drug resistance development in tumors and represent crucial focal points for tumor therapy and mechanisms underlying drug resistance. Incorporating herbal medicines as a complementary therapeutic approach to alleviate the toxicity and adverse effects associated with chemotherapy may improve the quality of life and survival of patients with cancer (Ye et al. 2015). Furthermore, increasing evidence suggests that *G. lucidum* could potentially overcome drug tolerance in tumors. Wu et al. (2012) extracted and purified *G. lucidum* spore oil and observed a gradual increase in its anticancer activity. Oil extracted after 1 h exhibited the most potent induction of cancer cell death. Furthermore, they demonstrated that miR‐378 overexpression enhanced cell survival and colony formation and conferred tolerance to multiple drugs. Ergosterol peroxides obtained from *G. lucidum* spore oil can eliminate chemotherapy‐resistant tumor cells mediated by miRNA‐378. Furthermore, cells that exhibit high resistance to chemotherapy drugs are also susceptible to *G. lucidum* oil‐induced cell death. Zhao, Ye, et al. (2011) reported that *G. lucidum* can induce cell cycle arrest in the G2/M phase and trigger apoptosis by activating caspase‐3, which effectively inhibited the growth and survival of several chemotherapy‐resistant epithelial ovarian cancer cell lines. Permeable glycoprotein (P‐gp) is a transmembrane drug transporter that can reduce intracellular drug levels, lower bioavailability, and induce the overexpression of some antitumor drugs (Alfarouk et al. 2015). This mechanism may be due to the large number of hydrogen bond donor groups and π‐electron rings (such as phenyl or tryptophan rings) that can form hydrogen bonds and induce π–π stacking and π–cation interactions with the drug, allowing P‐gp to attract the drug in the lipid environment and pump it out when water is available (Seelig 2020) (Figure 5). Furthermore, P‐gp leads to immunosuppression and, eventually, immune evasion. *G. lucidum* extract can inhibit P‐gp function and decrease the efflux of chemotherapeutic drugs, thus ensuring their efficacy. Similarly, Wu et al. (2022) found that ganoderiol F inhibited P‐gp transport activity but did not alter P‐gp expression. Moreover, they observed increased DOX accumulation in the human oral epidermoid carcinoma (KBv200) cell line, and that P‐gp‐mediated multidrug resistance was reversed. GLTs may contain potential reversal agents against multidrug resistance in tumors. Li, Cao, et al. (2023) found six extracts derived from *G. lucidum* with no obvious toxicity to cells and demonstrated that a *G. lucidum* ethanol extract could reverse resistance to DOX and paclitaxel by inhibiting P‐gp function in vitro. They also found that the *G. lucidum* ethanol extract could reverse resistance to paclitaxel in HepG‐2/ADM tumor‐bearing mice in vivo, but P‐gp activity and expression levels showed no significant change. This study revealed the effects of *G. lucidum* extract on multidrug resistance in human HCC cells and confirmed that the ethanol extract could reverse multidrug resistance by inhibiting P‐gp function both in vitro and in vivo. Not only can this compound reduce tumor drug resistance, but when combined with other drugs, it may increase the sensitivity of cancer cells to these drugs.

### 3.6 Telomerase Activity Inhibition

As telomeres play an important role in the life cycle of cancers, telomerase‐ and telomere‐based therapeutics may serve as alternatives to conventional treatments in the future (Ivancich et al. 2017). Telomerase is a complex of ribonucleic acid and protein and is a special DNA polymerase that uses part of its RNA as a template to synthesize and attach telomeric DNA to the ends of chromosomes (Schrumpfová and Fajkus 2020). Telomerase activity is absent in most normal human somatic cells, but more than 90% of cancer cells and in vitro‐immortalized cells exhibit telomerase activity (Roake and Artandi 2020). Once telomerase is activated, cells can become immortal and malignant (Hahn and Meyerson 2001). Malignant transformation of somatic cells may be caused by telomerase activation; therefore, inhibiting telomerase activity can effectively prevent the development of malignant tumors (Trybek et al. 2020). Furthermore, telomerase has received increasing attention as a tumor marker and therapeutic target (Kamal et al. 2020). The active compounds of *G. lucidum* can inhibit telomerase activity in liver and lung cancer cells, as well as in leukemia cells in vitro. Similarly, Yuen et al. (2007) observed that concentrated *G. lucidum* extracts could inhibit telomerase activity, reduce the viability and growth of human urothelial cell lines, and induce apoptosis in cancer cells. In addition, the extract induced HUC‐PC cell apoptosis under oxidative stress and increased H_2_O_2_ production, possibly due to oxidative attacks on telomere repeats (TTAAGGG) that generated 8‐OHdG (in a dose‐dependent manner) and led to telomerase shortening. Ding et al. (2023) investigated GLT inhibitory effects and mechanisms on the growth and metastasis of HCC cells and found that inactivating telomerase and DNA topoisomerase could inhibit inflammation by reducing carcinogenic signaling and exert antiangiogenic activity by suppressing angiogenesis.

The antitumor mechanism of *G. lucidum* is multifaceted and involves multiple targets and pathways. It can directly inhibit tumor cell growth and proliferation by interfering with their cell cycle, inducing apoptosis, and inhibiting angiogenesis. It can also indirectly inhibit tumor growth by regulating the immune system and enhancing the immune response against tumors. The immunomodulating effect of *G. lucidum* polysaccharides is particularly noteworthy, as they can activate various immune cells and promote cytokine production, thereby creating an unfavorable environment for tumor cell survival and proliferation. With the continuous research advancement on the active ingredients and antitumor mechanism of *G. lucidum* , more effective and safe antitumor drugs will be developed, which can contribute to the improvement of cancer treatment outcomes.

## 4 Conclusions and Perspectives

*G. lucidum* has exhibited notable anticancer activity in both in vitro studies and animal models; however, clinical trial data on its effectiveness in human cancer treatment remain relatively limited. This lack of information creates major uncertainty regarding the specific application and efficacy assessment of *G. lucidum* in cancer therapy. The fungus contains various active components, such as GLP and GLS, which exhibit high biological activity, yet the full understanding of their anticancer mechanisms remains elusive. This uncertainty limits the precise application and efficacy prediction of *G. lucidum* in cancer treatment.

The levels of active components in *G. lucidum* are influenced by a range of factors, including cultivation environment, extraction processes, and species variation. Ensuring the consistency and stability of these compounds in products is a critical area of ongoing research that requires further attention. Moreover, cancer patients demonstrate considerable individual variability in their *G. lucidum* sensitivity and response. Developing personalized treatment plans tailored to individual conditions and accurately assessing the anticancer efficacy of *G. lucidum* represent key research gaps that must be addressed.

With advancements in modern technology and deeper research into the active components of *G. lucidum* , future studies on its anticancer effects will focus on the following directions: (1) Exploring the cellular and molecular action mechanisms of *G. lucidum* active components to discover new anticancer targets and provide a theoretical basis for the development of novel anticancer drugs. Simultaneously, based on these anticancer mechanisms, various novel anticancer drugs will be developed. (2) Continuously optimizing extraction and purification techniques to improve the stability and bioavailability of *G. lucidum* active components, thereby ensuring product quality and efficacy. In the future, research on the anticancer effects of *G. lucidum* will emphasize interdisciplinary collaboration across biology, medicine, pharmacy, chemistry, and related fields. This integration of disciplines will advance studies on the anticancer mechanisms of *G. lucidum* and contribute to the development of more effective cancer therapies, with the ultimate goal of overcoming cancer.

## Author Contributions

Writing: Yuan Liu and Sizhu Ren. Data curation: Qing Sang and Xi Cheng. Supervision, project administration and funding acquisition: Yanmeng Bi. All authors have read and agreed to the published version of the manuscript.

## Ethics Statement

The authors have nothing to report.

## Conflicts of Interest

The authors declare no conflicts of interest.

## Data Availability

The authors have nothing to report.
